# Supplementary material for: Content-rich biological network constructed by mining PubMed abstracts
Source: BMC Bioinformatics. 2004 Oct 8;5:147. doi: 10.1186/1471-2105-5-147 (PMC528731; doi:10.1186/1471-2105-5-147)
Supplement: Additional File 2 — The original results of the above study (non-essential files are deleted to keep the file size under the limit set by BMC bioinformatics). [file 1471-2105-5-147-S2.bz2 › chilibotAdditionalFile2/dip05/98ID7768895E416/html/SNAP25_STX4A.html]

 


 **SNAP25** and **STX4A** 
  
Found 24 abstracts in PubMed, retrieved 05.  
 

 What does Google say? 
 PDF only 
| .edu only 

---

**Interactive relationship** (e.g. stimulation, inhibition, etc)

**Non-interactive relationship** (e.g. studied together, co-existance, homology, etc.)

- A combination of reverse transcription polymerase chain reaction and northern and western blotting is used to show expression of the target membrane SNARE, proteins SNAP 25  [ **SNAP25** ]  and syntaxin 4  [ **STX4A** ]  and the vesicular SNARE protein VAMP, the minimum molecular requirements for core exocytotic complex formation.  Ref: 11472886 Bone, 2001
